# Amygdala dismantled: the role of amygdala subregions in epilepsy

**DOI:** 10.1093/braincomms/fcad034

**Published:** 2023-02-17

**Authors:** Richard Zubal, Marian Galovic

**Affiliations:** Department of Neuroradiology, Inselspital, University Hospital Bern, Bern 3010, Switzerland; Department of Neurology, Cantonal Hospital St. Gallen, St. Gallen 9007, Switzerland; Department of Neurology, Clinical Neuroscience Centre, University Hospital and University of Zurich, Zurich 8091, Switzerland

## Abstract

This scientific commentary refers to: ‘Amygdala subnuclear volumes in temporal lobe epilepsy with hippocampal sclerosis and in non-lesional patients’ by Ballerini *et al*. (https://doi.org/10.1093/braincomms/fcac225).

**This scientific commentary refers to: ‘Amygdala subnuclear volumes in temporal lobe epilepsy with hippocampal sclerosis and in non-lesional patients’ by Ballerini *et al*. (https://doi.org/10.1093/braincomms/fcac225)**.

Modern imaging in epileptology has been looking beyond the obvious. In this regard, the obvious is hippocampal sclerosis, the hallmark of mesial temporal lobe epilepsy that can be easily spotted visually. A growing number of single-centre and multi-centre studies showed that structural changes in epilepsy are widespread, typically bilateral, and affect regions beyond the so-called epileptic focus.^1^ In the era of good-quality imaging and increasingly accurate computational neuroimaging analyses scientists are able to discover these changes *in vivo*. If proven to be robust, they could provide valuable information in stratifying patients with epilepsy, both in the diagnostic work-up and therapy.

Recently in *Brain Communications*, Ballerini *et al*.^2^ add further evidence to the above by ‘dismantling’ the amygdala. Previous studies^3,4^ already demonstrated trophic changes of the amygdala as a whole, in patients with temporal lobe epilepsy (TLE). Using modern parcellation methods the authors searched for more subtle changes on the level of amygdalar subregions. They found focal alterations of the ipsilateral amygdala in TLE with and without hippocampal sclerosis. The directionality of these changes differed, however. While cases with hippocampal sclerosis showed atrophy of the amygdala as a whole and of the basolateral complex in particular, cases without hippocampal sclerosis showed hypertrophy of the medial nucleus. The medial nucleus was also hypertrophic in extratemporal epilepsy, where this change was localised contralaterally to the epileptic focus. The subregional amygdalar alterations could discriminate between brains of people with epilepsy and healthy volunteers. Albeit the discriminative accuracy was modest, it could still prove an interesting marker in otherwise MRI negative cases.

These observations highlight the role of the amygdala as a subcortical circuit hub that is common to several types of epilepsies. They appear to, at least partially, question previous findings of epilepsy with amygdala enlargement representing a distinct TLE entity.^5^ The results of the current study propose that hypertrophy of the amygdala can be an epiphenomenon of temporal or extratemporal epilepsies. While amygdala hypertrophy could point towards an epilepsy associated tumour, hypertrophy can apparently also occur in cases without the diagnosis of a temporal tumour or in those with extratemporal epilepsy. Thus, isolated hypertrophy of the amygdala without any other imaging signs of an underlying tumour could represent a mere circuit phenomenon in epilepsy. It does not necessarily point towards a mesiotemporal onset, as hypertrophy was also observed in extratemporal cases. And it does not necessarily lateralise the epileptic focus, as it was observed contralaterally in extratemporal cases.

In comparison to the ipsilateral changes, such as hippocampal sclerosis, the affection of the contralateral amygdala may resemble the consequences rather than the cause of epilepsy. This makes such changes valuable in studying the epileptic disease process. Analysing the volumetric changes of the mesial temporal lobe structures on a subnuclear level may further help in understanding the pathophysiological changes in this network disease.

The amygdala has initially received some attention in the field of psychiatry, where amygdala volume enlargement was identified in people with psychosis. Ballerini and colleagues^2^ also found the association of amygdala hypertrophy affecting the ipsilateral lateral nucleus with psychiatric comorbidities. Although the exact mechanisms underlying these changes are not understood, they highlight the importance of the amygdala as an affective processing hub.

Another clinically important endeavour may be the correlation of amygdalar subregions with the risk for sudden unexpected death in epilepsy (SUDEP). Trophic changes of the amygdala as a whole have been previously associated with increased risk of SUDEP.^6^ Recently, neuropeptide depletion in the lateral nucleus of the amygdala has been postulated to be associated with SUDEP^7^ in a post mortem study without volumetric analysis. Perhaps an in vivo correlation of such finding could aid clinicians is stratifying the risk of their patients.

The current study by Ballerini and colleagues^2^ has limitations. While the subregional amygdala segmentation is clearly an advance, its validity in people with epilepsy still needs to be established. The authors cannot provide mechanistic insights on the pathophysiology underlying their findings and in this regard, correlation with functional and molecular imaging and connectivity measures may be helpful. When and how the changes arise also remains unclear. This highlights the need for longitudinal studies. It is also unknown, if the structural alterations are reversible, e.g. if they persist after successful epilepsy surgery.

The work of Ballerini *et al*.^2^ adds important building stones to modern epilepsy neuroimaging research on top of its established foundations. Dismantling the amygdala into its subregions, allowed for the recognition of focal changes that are hidden for visual analysis. Considering the complex functions and connections of specific amygdalar nuclei, making correlations on the nuclear level is a major advantage. Morphological biomarkers are in essence an available, inexpensive and noninvasive resource. In time biomarkers such as the trophic changes of the amygdala may find their way into clinical decision making.
